# Teachers’ self-regulated learning profiles in artificial intelligence contexts: implications for differentiated professional development

**DOI:** 10.3389/fpsyg.2026.1885895

**Published:** 2026-10-07

**Authors:** Yuhao Su, Mustafa Alpsülün, Müge Vuran, Hasan Celal Balıkçı, Müzeyyen Barut

**Affiliations:** 1 Faculty of Education, Henan University, Kaifeng, China; 2 Department of Computer Programming, Siverek Vocational School, Harran University, Şanlıurfa, Türkiye; 3 Ministry of National Education of the Republic of Türkiye, Ankara, Türkiye; 4 Institute of Educational Sciences, Fırat University, Elazig, Türkiye

**Keywords:** AI competency, artificial intelligence in education, person-centered analysis, self-regulated learning, teacher professional development, teacher professional learning

## Abstract

**Introduction:**

The rapid integration of artificial intelligence (AI) into education requires teachers to develop new competencies and manage their own learning. While research has examined teachers’ AI attitudes, acceptance, and literacy, less is known about how they regulate their learning in AI contexts. This study examined teachers’ self-regulated learning (SRL) profiles and their associations with demographic characteristics and AI-related experience.

**Methods:**

In a descriptive survey, 213 in-service teachers completed the Artificial Intelligence Self-Regulated Learning Scale for Teachers (AI-SRLS). Two-Step Cluster Analysis was conducted using five dimensions: task planning, self-monitoring, help seeking, institutional support, and self-evaluation.

**Results:**

Two profiles emerged: High Strategic Learners, who scored consistently higher across all five dimensions, and Limited Strategy Users, who reported comparatively lower strategy use. Profile membership was significantly associated with prior AI experience and AI-related training. Gender, age, and professional experience did not significantly differentiate the profiles.

**Discussion:**

Teachers’ AI-related SRL strategies co-occur in distinct profiles, supporting a differentiated approach to professional development. Teachers with limited strategy use may benefit from structured guidance, scaffolded AI learning opportunities, and stronger institutional support, while highly strategic learners may benefit from more autonomous and advanced learning opportunities.

## 1 Introduction

The rapid development of artificial intelligence (AI) is reshaping educational practice and increasing demands on teachers to continuously develop new technological and pedagogical competencies. Although AI is defined in various ways, it is generally conceptualized as systems capable of performing cognitive functions traditionally associated with human intelligence (Temur, 2025). The widespread adoption of these technologies is not only altering technological infrastructures but also reshaping how individuals learn, solve problems, and communicate in both personal and professional contexts.

Education is among the fields where the impact of AI has become particularly prominent. AI-based applications are increasingly being integrated into educational settings to support learning processes, create adaptive and interactive learning environments, and identify learners’ needs more accurately (Hwang et al., 2020). These technologies can support teachers in areas such as lesson planning, assessment and feedback, instructional resource development, and other teaching-related tasks (Sumandal, 2023; Tan et al., 2025a). At the same time, teachers’ perceptions of AI, their levels of AI literacy, and their tendencies toward technology use have emerged as critical variables in educational research (Kohnke et al., 2025; López-Costa et al., 2025).

Despite these opportunities, integrating AI into educational practice is not a straightforward process. It requires teachers to continuously update their knowledge, adapt to rapidly evolving technologies, and make informed pedagogical decisions under conditions of uncertainty. In this respect, learning to use AI can be conceptualized as an ongoing, self-directed professional learning process rather than a one-time acquisition of technical skills.

Self-regulated learning (SRL) provides a robust theoretical framework for understanding such processes. SRL refers to the integration of cognitive, motivational, and behavioral processes that enable individuals to actively manage their own learning. Core components of SRL include setting learning goals, selecting and implementing appropriate strategies, monitoring progress, and evaluating outcomes (Kauffman, 2004). In AI-supported learning environments, the importance of SRL becomes even more pronounced, as teachers are increasingly required to independently acquire new competencies, regulate their learning processes, and respond to emerging instructional challenges.

However, despite the growing body of research on AI in education, studies examining teachers’ strategic learning behaviors within AI contexts remain limited. Existing research, particularly in the Turkish context, has primarily focused on teachers’ metaphorical perceptions of AI, technology acceptance levels, and AI literacy (Balıkçı et al., 2024; Balıkçı and Alpsülün, 2025). While these studies provide valuable insights into teachers’ attitudes and readiness, they offer limited understanding of how teachers regulate their own learning processes when engaging with AI technologies. In particular, relatively little research has examined which self-regulation strategies teachers employ and how these strategies may combine into distinct learner profiles. This gap is critical, as understanding such profiles can provide deeper insights into teachers’ professional development needs and inform the design of targeted educational policies and interventions.

SRL theory further emphasizes that learning unfolds through cyclical phases of planning, performance, and self-reflection (Zimmerman and Moylan, 2009). In the context of teachers’ AI learning, task planning involves defining learning goals and organizing learning activities; self-monitoring requires the regulation of cognitive processes and problem-solving strategies; help seeking includes drawing on peer and institutional resources; institutional support reflects access to training, infrastructure, and guidance; and self-evaluation enables teachers to assess their progress and refine their learning strategies. These dimensions collectively provide a comprehensive framework for analyzing teachers’ engagement in AI learning processes.

Previous research on teachers’ engagement with artificial intelligence has predominantly examined factors such as AI perceptions, technology acceptance, AI literacy, perceived usefulness, self-efficacy, and institutional support. Collectively, these studies have provided important insights into teachers’ readiness to adopt AI and the individual and contextual factors associated with its acceptance and use (Balıkçı et al., 2024; Balıkçı and Alpsülün, 2025; Al Darayseh, 2023; Ma and Lei, 2024; Cabero-Almenara et al., 2024). A recent systematic review further indicates that research has devoted substantially greater attention to the application of AI in teaching than to teachers’ professional development, highlighting the need for a stronger focus on how teachers develop the capacities required for effective AI integration (Tan et al., 2025a). However, this literature has largely approached teachers as a relatively homogeneous group and has focused on whether teachers are prepared or willing to engage with AI rather than on how they strategically regulate their own learning while developing AI-related competencies.

Consequently, little is known about whether distinct groups of teachers exhibit different configurations of self-regulated learning strategies in AI learning contexts. In particular, previous research provides limited evidence regarding how task planning, self-monitoring, help seeking, institutional support, and self-evaluation operate together within individual teachers and whether different combinations of these strategies characterize meaningful learner profiles. This represents an important gap because variable-centered analyses of individual factors may obscure heterogeneity among teachers and, consequently, provide limited guidance for designing professional development that responds to different learning needs.

To address this gap, the present study adopts a person-centered approach to identify distinct teacher profiles based on five dimensions of AI-related self-regulated learning: task planning, self-monitoring, help seeking, institutional support, and self-evaluation. It further examines whether membership in these profiles differs according to demographic characteristics and AI-related experience and training. The novelty of the study lies in shifting the focus from isolated indicators of AI readiness toward the configurations in which multiple self-regulated learning dimensions co-occur within teachers. By identifying heterogeneous SRL profiles, the study provides a basis for understanding differentiated professional learning needs and for designing more targeted professional development in AI-integrated educational contexts.

## 2 Theoretical framework

### 2.1 Teachers’ professional development in the age of artificial intelligence: a self-regulated learning-based perspective

The rapid advancement of artificial intelligence (AI) technologies has fundamentally transformed educational systems and redefined the nature of teaching as a profession. Beyond merely introducing new technological tools, AI has reshaped instructional processes by enabling adaptive learning environments, automated assessment systems, real-time feedback mechanisms, and data-driven decision making. These developments have significantly increased expectations regarding teachers’ digital pedagogical competencies and their ability to integrate AI into instructional practices effectively (Lindner and Romeike, 2019; Guan et al., 2025).

In this evolving landscape, teachers are no longer positioned solely as knowledge transmitters but as facilitators, designers, and critical evaluators of AI-supported learning environments. Consequently, teachers are required to continuously update their knowledge, interpret emerging technologies, and make pedagogically sound decisions under conditions of uncertainty. This transformation highlights that teacher learning in AI contexts is not episodic but continuous, dynamic, and self-directed. Recent evidence further suggests that teachers’ AI competence is multidimensional and requires continuous professional learning rather than one-time technical training. Delcker et al. (2025), for example, found substantial variation across teachers’ AI competence dimensions and emphasized the need for ongoing professional learning opportunities that address both AI-specific competencies and continuing professionalization.

International reports and related research further emphasize the urgency of this transformation. For instance, OECD (2023) and UNESCO (2023) emphasize the importance of systematic professional development for strengthening teachers’ digital literacy and AI-related pedagogical competencies. In this context, variables such as teachers’ perceptions of AI, self-efficacy beliefs, and behavioral intentions play a decisive role in technology integration processes (Daher, 2025; Al Darayseh, 2023).

Given these demands, teachers’ ability to regulate their own learning processes becomes a critical component of effective professional development. Therefore, self-regulated learning (SRL) offers a powerful theoretical lens through which teachers’ AI learning processes can be examined.

### 2.2 Self-regulated learning: theoretical foundations

Self-regulated learning is grounded in metacognitive theory and emphasizes individuals’ capacity to actively control and regulate their cognitive, motivational, and behavioral processes during learning (Zelazo, 2014; Schunk and Zimmerman, 2011). Within this framework, learners are conceptualized as proactive agents who set goals, select appropriate strategies, monitor their progress, and evaluate outcomes.

One of the most influential models in SRL literature is Zimmerman’s cyclical model, which conceptualizes learning as an iterative process consisting of three interrelated phases:

- *Forethought phase*: Goal setting, strategic planning, and motivational beliefs.
- *Performance phase*: Strategy implementation, self-monitoring, and behavioral control.
- *Self-reflection phase*: Self-evaluation and adaptive regulation.

These phases are not linear but dynamically interconnected, allowing learners to continuously refine their strategies based on feedback and experience (Zimmerman and Moylan, 2009).

From a professional development perspective, SRL is particularly relevant for teachers, as it enables them to manage their own learning processes in complex and evolving environments. Research indicates that teachers who effectively use SRL strategies demonstrate higher levels of professional growth, instructional effectiveness, and adaptive expertise (Randi, 2004; Greene et al., 2023). These strategies include goal setting, reflective practice, help seeking, and the effective use of institutional resources.

Zimmerman’s cyclical SRL model serves as the primary theoretical foundation for understanding self-regulated learning in the present study. The five dimensions examined in the empirical analysis, namely Task Planning, Self-Monitoring, Help Seeking, Institutional Support, and Self-Evaluation, constitute the operational structure adopted in this study rather than five original components of Zimmerman’s model. These dimensions are therefore used to operationalize individual regulatory processes and contextual conditions relevant to teachers’ AI-related professional learning within the broader theoretical perspective provided by Zimmerman’s cyclical model.

### 2.3 Teachers’ self-regulated learning in artificial intelligence contexts (AI-SRL)

The integration of AI into education introduces new forms of complexity that intensify the need for self-regulation in teachers’ learning processes. Unlike traditional technologies, AI systems are often characterized by opacity, rapid evolution, and context-dependent functionality. As a result, teachers are required not only to learn how to use AI tools but also to critically evaluate their pedagogical implications and adapt them to diverse instructional contexts.

Taken together, existing research suggests that teachers’ engagement with AI is shaped not only by their knowledge of AI technologies but also by their confidence, opportunities for learning, and capacity to respond to uncertainty and rapidly changing technological demands (Pokrivcakova, 2023; Hwang et al., 2020; Chounta et al., 2022). From an SRL perspective, these challenges are particularly relevant because effective professional learning requires teachers to set learning goals, monitor their developing understanding, seek assistance when needed, and evaluate their progress as they engage with unfamiliar AI tools and practices.

Research on teachers’ technology adoption further indicates that engagement with AI is associated with factors such as perceived usefulness, perceived ease of use, self-efficacy, institutional support, and digital pedagogical beliefs (Gökçe Tekin, 2024; Al Darayseh, 2023; Cabero-Almenara et al., 2024; Ma and Lei, 2024). Collectively, this literature provides an important account of the individual and contextual conditions associated with teachers’ willingness and readiness to engage with AI.

However, while this literature helps explain the conditions associated with whether teachers accept or engage with AI, it provides comparatively limited insight into how teachers regulate the learning processes through which they develop AI-related competencies. This distinction is theoretically important because willingness or readiness to adopt AI does not, by itself, explain how teachers plan, monitor, seek support for, and evaluate their continuing professional learning.

Therefore, examining teachers’ AI-related professional learning through an SRL perspective provides a useful framework for understanding how teachers actively manage their learning in rapidly evolving AI contexts. In the present study, AI-related SRL is understood as the application of self-regulatory processes within technology-mediated and context-sensitive professional learning environments. Recent research has begun to conceptualize AI literacy itself through a self-regulatory lens. For example, Kaya and Meylani (2026) operationalized AI-related learning through dimensions including metacognitive monitoring, motivational regulation, behavioral regulation, and critical evaluation, further illustrating the relevance of regulatory processes for learning effectively with AI.

Taken together, the increasing demands of AI-related professional learning highlight the importance of SRL processes through which teachers plan, monitor, and evaluate their learning, while institutional support provides the contextual resources and opportunities that may enable these processes to operate effectively.

### 2.4 Conceptual framework: from AI-SRL strategies to teacher profiles

Building on the theoretical foundations of SRL and the emerging literature on AI in education, the present study conceptualizes teachers’ AI-related professional learning through five interconnected dimensions: task planning, self-monitoring, help seeking, institutional support, and self-evaluation.

- *Task planning*: Defining AI-related learning goals and organizing learning activities.
- *Self-monitoring*: Tracking progress and regulating cognitive processes during AI learning.
- *Help seeking*: Utilizing peer, expert, and institutional support mechanisms.
- *Institutional support*: Accessing organizational resources, training, and infrastructure.
- *Self-evaluation*: Reflecting on learning outcomes and adjusting strategies accordingly.

While task planning, self-monitoring, help seeking, and self-evaluation represent individual regulatory processes, institutional support captures the contextual resources and conditions within which teachers regulate their AI-related professional learning. Accordingly, the five dimensions reflect both individual regulatory processes and the contextual conditions that may support teachers’ professional learning in AI-related contexts.

Conceptually, these five dimensions should not be viewed as isolated indicators. Rather, they represent interconnected individual and contextual dimensions of teachers’ AI-related professional learning. Task planning reflects the forethought and organization of AI-related learning goals, whereas self-monitoring captures the ongoing regulation of learning activities. Help seeking represents teachers’ strategic use of interpersonal resources, while institutional support reflects the contextual opportunities and resources available for sustained professional learning. Self-evaluation, in turn, represents reflective judgments through which teachers assess learning outcomes and adapt subsequent learning strategies. Together, these dimensions capture cognitive, behavioral, reflective, and contextual aspects of AI-related professional learning.

Building on this multidimensional structure, the present study adopts a person-centered perspective. Rather than examining each SRL dimension independently, this perspective considers how the five dimensions combine within individuals to form distinct configurations of strategy use. This distinction is theoretically important because teachers with similar scores on one SRL dimension may differ substantially in their overall patterns of planning, monitoring, help seeking, perceived institutional support, and self-evaluation. Accordingly, examining configurations of these dimensions can reveal heterogeneity that may remain obscured when relationships are examined only at the variable level.

The conceptual framework of the study therefore proposes that the joint configuration of task planning, self-monitoring, help seeking, institutional support, and self-evaluation can be used to identify distinct teacher SRL profiles in AI learning contexts (Figure 1). These profiles are subsequently examined in relation to demographic characteristics, AI experience, and prior AI-related training. This framework connects SRL theory with a person-centered analytical perspective and provides the conceptual basis for examining differentiated professional learning needs in AI-integrated educational environments.

**Figure 1:**
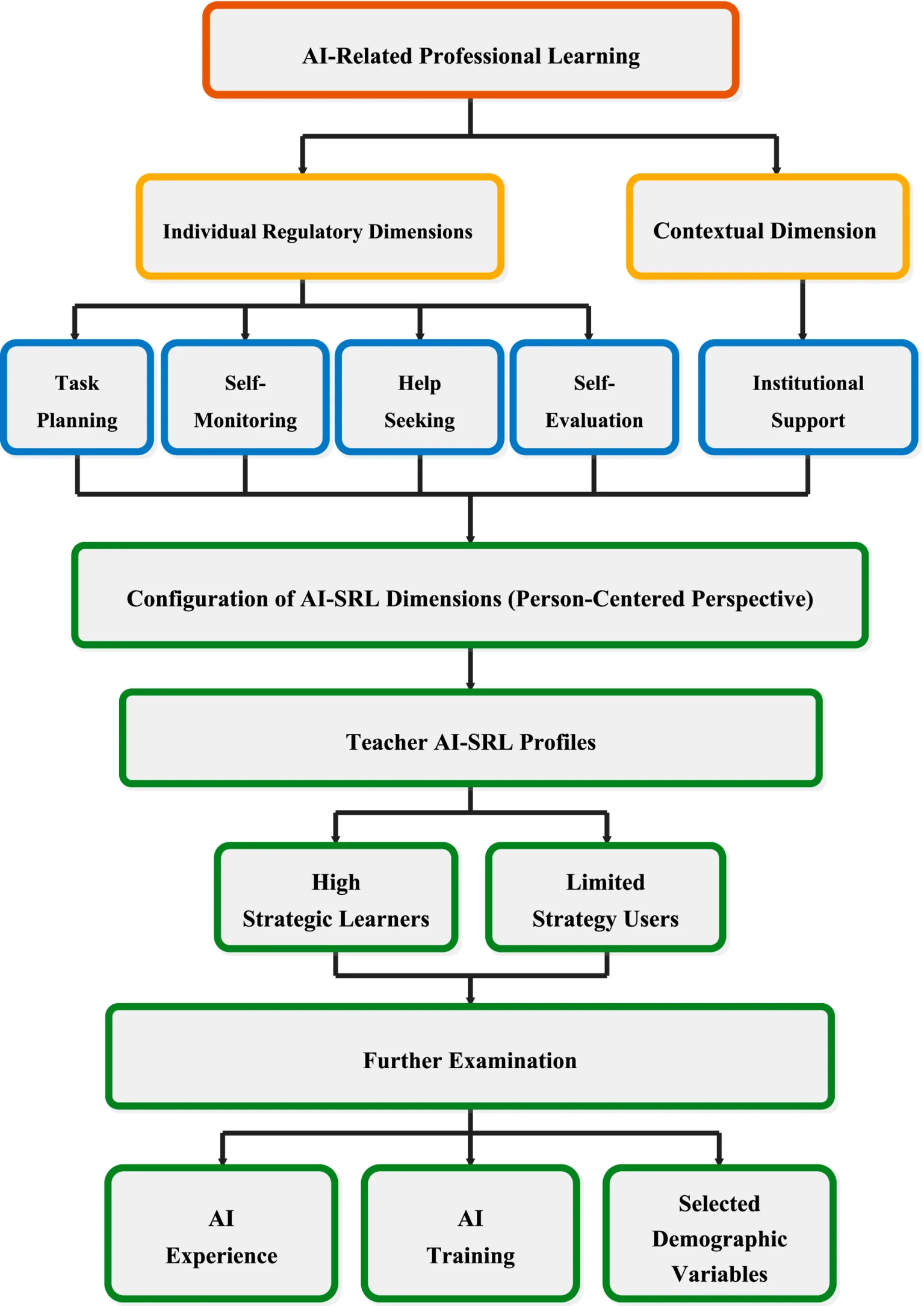
Conceptual framework linking individual and contextual dimensions of AI-related self-regulated professional learning to teacher SRL profiles.

Accordingly, Institutional Support is conceptualized not as an individual self-regulatory strategy, but as a contextual condition that provides the resources and opportunities within which individual regulatory processes can operate.

## 3 Methods

### 3.1 Research design

This study was designed using a descriptive survey model to examine the self-regulated learning (SRL) strategies employed by teachers in artificial intelligence (AI) learning processes and to identify distinct profiles based on these strategies. Descriptive survey designs are widely used in educational research to systematically describe existing phenomena and to capture patterns within large samples (Creswell and Creswell, 2017).

The study primarily adopted a person-centered analytical approach to identify heterogeneous profiles of teachers’ AI-related self-regulated learning and subsequently examine their associations with selected demographic and AI-related characteristics. Person-centered approaches provide a deeper understanding of heterogeneity within a population and allow researchers to uncover meaningful patterns that may remain hidden in traditional variable-centered analyses (Bergman and Magnusson, 1997; Howard and Hoffman, 2018). In the context of teacher learning, such approaches are particularly valuable for revealing differences in how individuals regulate their learning processes.

Within this framework, teachers’ profiles based on AI-related self-regulated learning strategies were identified using Two-Step Cluster Analysis. Cluster analysis is an exploratory multivariate technique that aims to classify individuals into relatively homogeneous groups while maximizing differences between clusters (Hair et al., 2019). Given the exploratory objective of the study and the aim of obtaining an interpretable empirical classification, Two-Step Cluster Analysis was selected as the primary analytical approach.

By employing a person-centered clustering approach, this study moves beyond examining average tendencies and instead focuses on identifying distinct strategy profiles among teachers. This design is particularly appropriate for understanding complex learning behaviors in AI contexts, where individual differences in strategy use play a critical role.

### 3.2 Participants

The study group consisted of 213 in-service teachers working in different provinces of Türkiye. Participants were recruited through professional teacher networks and online communication channels commonly used by teachers, and participation was entirely voluntary. The inclusion criteria required participants to be currently employed as in-service teachers and to provide informed consent to participate in the study. Responses from individuals who did not meet these criteria or who provided insufficient data to calculate the AI-SRLS subdimension scores were considered ineligible for analysis.

A total of 213 teachers participated in the study, and all 213 met the eligibility criteria and were retained in the final dataset. No participants were excluded from the analyses. Because the survey link was distributed through professional networks and online communication channels rather than to a predefined sampling frame with a known number of invited teachers, a conventional response rate could not be calculated.

The final sample included teachers with variation in subject areas, age groups, levels of professional experience, and school types, thereby allowing the study to examine potentially heterogeneous patterns of AI-related self-regulated learning. The sample size was considered suitable for the exploratory cluster analysis; however, the relatively small size of the second cluster (*n* = 40) limits the generalizability and reproducibility of the identified profile.

The demographic information collected from participants included gender, age range, teaching experience, school type, educational levels taught, experience in using artificial intelligence (AI), and prior AI-related training. These variables were used to examine differences between the identified clusters and to provide a more comprehensive interpretation of the resulting profiles (see Table 1).

**Table 1:** Selected demographic characteristics of participants.

Variable	Category	*n*	%
Gender	Female	118	55.4
	Male	95	44.6
AI experience	Yes	136	63.8
	No	77	36.2
AI training	Yes	82	38.5
	No	131	61.5

### 3.3 Data collection instrument

Data were collected using the Turkish version of the Artificial Intelligence Self-Regulated Learning Scale for Teachers (AI-SRLS), which was adapted by Alpsülün et al. (2025) based on the original scale developed by Zhang et al. (2025). The scale is designed to assess teachers’ use of self-regulated learning (SRL) strategies in artificial intelligence (AI)-supported learning processes and provides a multidimensional measurement of strategic learning behaviors.

The adapted scale consists of 30 items grouped under five theoretically grounded dimensions:

1. Task planning.
2. Self-monitoring.
3. Help seeking.
4. Institutional support.
5. Self-evaluation.

These dimensions are aligned with the core components of self-regulated learning theory and reflect the cognitive, behavioral, and contextual aspects of teachers’ learning processes in AI environments.

Previous validation studies of the Turkish form have demonstrated that the scale possesses strong psychometric properties. Confirmatory factor analysis (CFA) supported the five-factor structure, indicating an acceptable model fit (CFI = 0.951; RMSEA = 0.057). In addition, internal consistency coefficients were high across all dimensions, with Cronbach’s alpha values ranging from 0.86 to 0.93, indicating a high level of reliability.

All items were rated on a five-point Likert scale ranging from 1 (strongly disagree) to 5 (strongly agree). For the purposes of this study, mean scores were calculated for each subdimension rather than using total scores, in order to preserve the multidimensional structure of the construct. These subdimension scores were subsequently used as input variables in the cluster analysis.

The use of a multidimensional SRL scale is particularly appropriate in person-centered analyses, as it allows for the identification of distinct profiles based on combinations of strategy use rather than overall levels. In this context, the AI-SRLS provides a comprehensive and theoretically grounded measurement tool for examining teachers’ strategic learning behaviors in AI-supported environments.

### 3.4 Data collection procedure

Data were collected through an online survey administered to in-service teachers across different regions of Türkiye. The use of an online data collection method was preferred in order to facilitate access to a diverse sample and to ensure efficiency in reaching participants from various geographical locations.

Participation in the study was entirely voluntary. Prior to completing the survey, participants were provided with a clear explanation of the purpose of the study, the scope of data collection, and the intended use of the data. Informed consent was obtained from all participants before data collection proceeded.

Ethical considerations were carefully addressed throughout the research process. Participants were assured that their responses would remain confidential and would be used solely for academic purposes. No personally identifiable information was collected, and all data were analyzed anonymously. These procedures were implemented in accordance with standard ethical guidelines for research involving human participants (Creswell and Creswell, 2017).

The data collection process was conducted over a specified period, during which the survey link was distributed through professional networks and online communication channels commonly used by teachers. This approach enabled the inclusion of participants with varying backgrounds and experiences related to artificial intelligence.

### 3.5 Data analysis

Data analysis was conducted in three sequential stages to ensure the validity, reliability, and interpretability of the findings.

#### 3.5.1 Preliminary analyses

Prior to the main analyses, several preliminary procedures were carried out to assess the suitability of the data for cluster analysis. First, the dataset was screened for missing values. No missing values were identified in the study variables; therefore, no imputation or case deletion procedure was required, and all 213 participants were retained for the analyses.

Descriptive statistics (means, standard deviations, minimum and maximum values) were then examined for all variables. Descriptive distributional characteristics were examined using skewness and kurtosis, and no severe deviations from normality were observed.

In addition, the internal consistency of the AI-SRLS subdimensions was assessed for the present sample using Cronbach’s alpha coefficients. The coefficients ranged from 0.73 to 0.97 across the five subdimensions, indicating acceptable to high internal consistency (Nunnally and Bernstein, 1994).

One negatively worded item in the Help Seeking dimension was reverse-coded prior to analysis. Following these procedures, the dataset was deemed suitable for subsequent cluster analysis.

#### 3.5.2 Two-step cluster analysis

To identify distinct profiles based on teachers’ AI-related self-regulated learning strategies, a Two-Step Cluster Analysis was conducted using the five AI-SRL subdimensions as clustering variables. The Two-Step method was selected because the primary aim of the study was to explore teacher profiles without specifying the number of clusters *a priori*. Unlike K-means clustering, which requires the number of clusters to be predetermined, the Two-Step procedure allows alternative cluster solutions to be evaluated using information criteria. Compared with conventional hierarchical clustering, it integrates pre-clustering and hierarchical grouping within a computationally efficient procedure (Hair et al., 2019; Norušis, 2011). Latent Profile Analysis (LPA) represents a model-based alternative for identifying latent subpopulations but requires additional distributional and model-specification decisions. Given the exploratory objective of the study, the sample size, the use of continuous indicators, and the aim of obtaining an interpretable empirical classification, Two-Step Cluster Analysis was considered appropriate for the present study.

Prior to clustering, scores for the five AI-SRL subdimensions were standardized as z-scores to eliminate scale differences and ensure that each dimension contributed comparably to the clustering process. The number of clusters was determined by considering the Bayesian Information Criterion (BIC) together with the Silhouette measure of cohesion and separation. BIC provides an information-based criterion for comparing candidate solutions, whereas the Silhouette coefficient indicates the extent to which cases are internally cohesive within their assigned clusters and separated from other clusters (Rousseeuw, 1987).

Alternative solutions ranging from two to five clusters were examined. The automatic Two-Step clustering procedure selected a two-cluster solution. The candidate solutions yielded BIC values of 610.048, 575.565, 583.049, and 594.784 for the two-, three-, four-, and five-cluster solutions, respectively. Although the three-cluster solution had the lowest absolute BIC value, the automatic Two-Step procedure selected the two-cluster solution based on the sequential changes in BIC and the ratio of distance measures. In addition, the two-cluster solution yielded the highest Silhouette coefficient (0.43), compared with 0.26, 0.26, and 0.23 for the three-, four-, and five-cluster solutions, respectively, indicating comparatively greater within-cluster cohesion and between-cluster separation (Kaufman and Rousseeuw, 1990). Considering the automatic model-selection procedure, comparative cluster quality, and substantive interpretability, the two-cluster solution was retained as the final model (see Table 2).

**Table 2:** Comparison of alternative two-step cluster solutions.

Cluster solution	BIC	Silhouette	Decision
2	610.048	0.43	Retained
3	575.565	0.26	Not retained
4	583.049	0.26	Not retained
5	594.784	0.23	Not retained

To further evaluate the robustness of the identified cluster structure, an additional K-means sensitivity analysis was conducted using the same five standardized AI-SRL dimensions. Solutions containing two to five clusters were compared using the Silhouette coefficient. Consistent with the primary Two-Step analysis, the two-cluster K-means solution produced the highest Silhouette coefficient (0.38), compared with 0.31, 0.28, and 0.29 for the three-, four-, and five-cluster solutions, respectively. The two-cluster K-means solution also produced a substantively similar pattern, distinguishing teachers with consistently higher AI-SRL scores from those with comparatively lower scores across the five dimensions. Although formal resampling-based stability testing was not conducted, alternative cluster solutions were compared and a K-means sensitivity analysis was performed as supplementary robustness checks. The K-means analysis yielded a substantively similar two-profile pattern, providing supplementary evidence for the robustness and interpretability of the retained solution rather than evidence of formal statistical stability.

Based on the final Two-Step model, each participant was assigned to a cluster. Cluster profiles were subsequently interpreted by examining their mean scores across task planning, self-monitoring, help seeking, institutional support, and self-evaluation. The clusters were labeled according to their overall patterns of strategy use.

#### 3.5.3 Comparison of clusters according to demographic variables

To examine whether the identified clusters differed significantly according to demographic variables, appropriate statistical tests were selected based on the type and distribution of the data.

- Chi-square tests were used for categorical variables (e.g., gender, AI experience, AI training) to examine differences in group distributions.
- For continuous variables, independent samples *t*-tests were applied when normality assumptions were met, while the Mann–Whitney U test was used as a non-parametric alternative when assumptions were violated.
- In cases involving more than two groups, one-way ANOVA was conducted.

In addition to statistical significance, effect sizes were reported to assess the magnitude and practical relevance of the observed differences (Cohen, 1988).

## 4 Findings

### 4.1 Descriptive statistics

The mean and standard deviation values for the five subdimensions of teachers’ self-regulated learning (SRL) strategies in artificial intelligence (AI) learning contexts are presented in Table 3.

**Table 3:** Descriptive statistics for the subdimensions.

Subdimension	Mean	Standard deviation	Minimum	Maximum
Task planning (TP)	4.15	0.89	1.00	5.00
Self monitoring (SM)	3.49	0.78	1.00	5.00
Help seeking (HS)	3.84	0.81	1.00	5.00
Institutional support (IS)	3.24	0.95	1.00	5.00
Self evaluation (SE)	3.45	0.84	1.00	5.00

Teachers reported relatively high levels of AI-related self-regulated learning overall, with Task Planning showing the highest mean score (M = 4.15, SD = 0.89). They also reported comparatively lower levels of perceived institutional support (M = 3.24, SD = 0.95) than the other AI-SRL dimensions. This finding reflects teachers’ perceptions of the contextual support available to them rather than an objectively established institutional deficit.

### 4.2 Two-step cluster analysis

A Two-Step Cluster Analysis was conducted to identify distinct groups of teachers based on similarities in their self-regulated learning (SRL) strategies in artificial intelligence (AI) learning contexts. As reported in the Data Analysis section, alternative solutions ranging from two to five clusters were compared using the BIC and Silhouette measures. The automatic Two-Step procedure selected the two-cluster solution, which also yielded the highest Silhouette coefficient (0.43) among the candidate solutions. Accordingly, the two-cluster solution was retained for subsequent profile interpretation and between-group analyses.

The analysis yielded a two-cluster solution as the most appropriate model. The Silhouette value was calculated as 0.43, indicating a moderate but acceptable level of cluster quality, with a meaningful degree of separation between clusters while allowing for some overlap. This level of cohesion and separation is considered sufficient for identifying interpretable group structures in social science research.

The distribution of participants across clusters is presented in Table 4.

**Table 4:** Cluster distributions.

Cluster	Frequency	Percentage (%)
Cluster 1	173	81.2
Cluster 2	40	18.8

The two-cluster solution showed an unequal distribution, with 173 teachers (81.2%) in Cluster 1 and 40 teachers (18.8%) in Cluster 2. The smaller cluster represents a less prevalent pattern of AI-SRL strategy use that may warrant greater attention in professional development design.

Overall, the clustering results provide evidence that teachers’ AI-related self-regulated learning behaviors are not homogeneous but can be meaningfully categorized into distinct profiles. This finding supports the suitability of a person-centered analytical approach for capturing variability in teachers’ learning strategies.

### 4.3 Characteristics of cluster profiles

The mean scores of teachers in each cluster across the five self-regulated learning (SRL) strategy dimensions are presented in Table 5.

**Table 5:** Subdimension means by cluster.

Subdimension	Cluster 1 (high strategic learners)	Cluster 2 (limited strategy users)
Task planning	4.55	2.39
Self monitoring	3.77	2.28
Help seeking	4.12	2.63
Institutional support	3.51	2.10
Self evaluation	3.76	2.09

The results reveal a clear and consistent separation between the two clusters across all five dimensions, indicating that the identified profiles differ not only in overall strategy use but also in the way individual self-regulation processes are enacted.

The two clusters differed consistently across all five AI-SRL dimensions. High Strategic Learners reported higher levels of task planning, self-monitoring, help seeking, institutional support, and self-evaluation than Limited Strategy Users. This pattern indicates that the profiles differ across the overall configuration of AI-SRL dimensions rather than on a single strategy 7 (see Figure 2).

**Figure 2:**
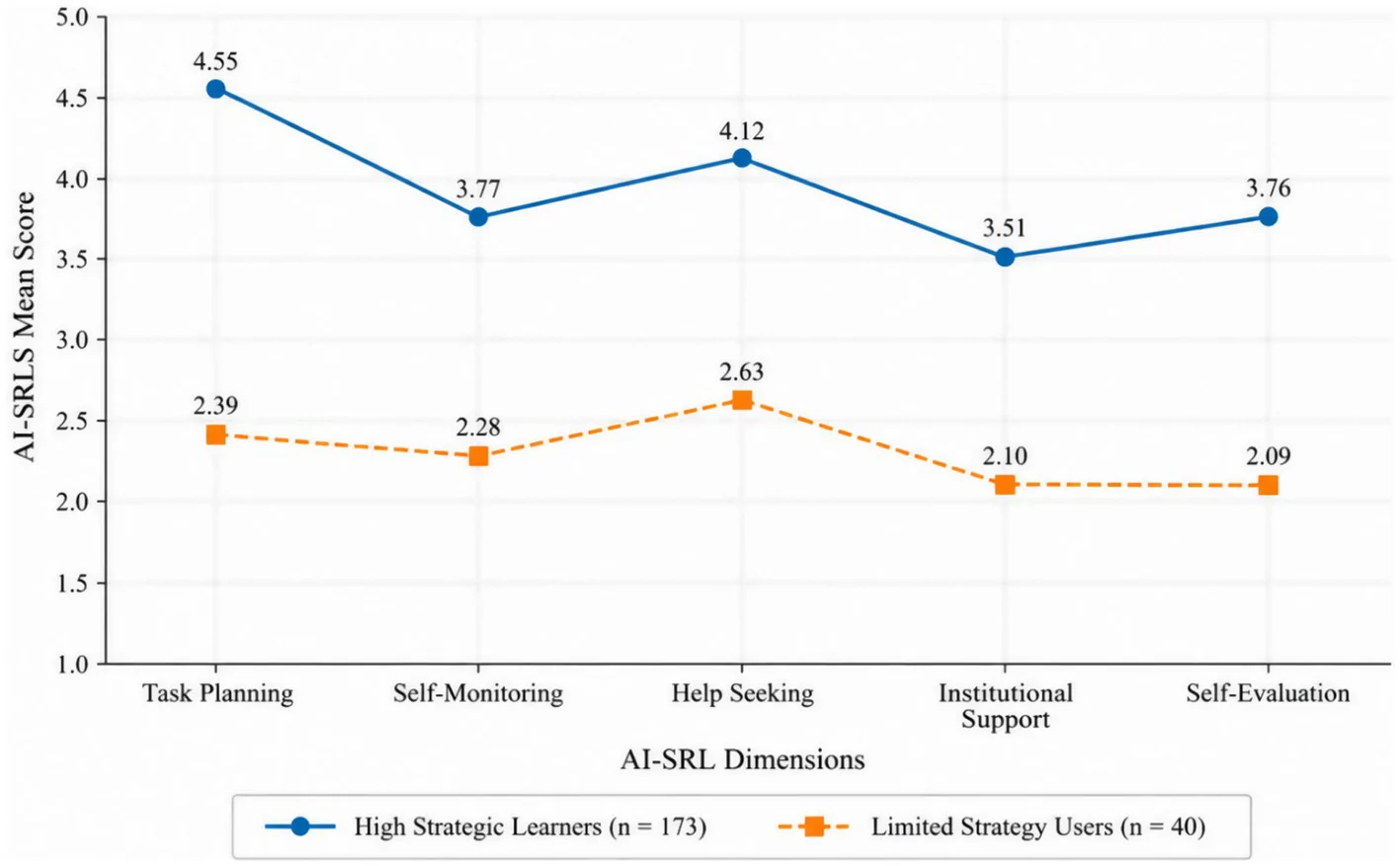
Mean AI-SRL dimension scores for high strategic learners and limited strategy users.

### 4.4 Comparison of clusters according to demographic and AI-related variables

To further characterize the identified profiles, cluster membership was examined in relation to demographic and AI-related variables. Chi-square tests were used for categorical variables, while appropriate parametric or nonparametric procedures were used for continuous variables. Effect sizes were reported where applicable to complement statistical significance and indicate the practical magnitude of the observed associations.

#### 4.4.1 AI experience

A statistically significant association was found between cluster membership and prior AI usage experience, χ^2^(1, *N* = 213) = 9.84, *p* = 0.002, Cramer’s V = 0.21. Teachers with prior AI experience were more likely to belong to the High Strategic Learners profile. The effect size indicates a small-to-moderate association, suggesting that practical exposure to AI is meaningfully related to how teachers regulate their AI-related professional learning.

#### 4.4.2 AI training

Cluster membership was also significantly associated with prior AI-related training, χ^2^(1, *N* = 213) = 11.27, *p* = 0.001, Cramer’s V = 0.23. Teachers who had received AI-related training were more frequently represented among High Strategic Learners. The magnitude of the association was small to moderate, indicating that structured AI learning opportunities are meaningfully associated with stronger configurations of self-regulated learning strategies.

#### 4.4.3 Gender

No statistically significant association was found between gender and cluster membership, χ^2^(1, *N* = 213) = 1.12, *p* = 0.290, Cramer’s V = 0.07. The small effect size, together with the nonsignificant result, indicates that gender provides little practical differentiation between the two AI-SRL profiles.

#### 4.4.4 Other demographic variables

No statistically significant differences in cluster membership were observed according to age, teaching experience, or school type (all *p* > 0.05). Effect sizes were not available for these comparisons; therefore, the findings should be interpreted on the basis of the available inferential statistics and with appropriate caution.

#### 4.4.5 Overall interpretation

Taken together, AI experience and prior AI-related training were significantly associated with profile membership, with small-to-moderate effect sizes, whereas gender showed a small and nonsignificant association. No statistically significant differences were observed for age, teaching experience, or school type.

## 5 Discussion

The identification of two AI-SRL profiles suggests that teachers’ professional learning is characterized by heterogeneous configurations of regulatory processes rather than a uniform pattern of strategy use. The distinction between High Strategic Learners and Limited Strategy Users further indicates that variation in AI-related professional learning reflects the co-occurrence of multiple regulatory dimensions rather than isolated differences in individual strategies.

The emergence of these two profiles is consistent with the cyclical and multidimensional nature of self-regulated learning. SRL frameworks conceptualize planning, monitoring, strategy adjustment, help seeking, and reflection as interconnected processes through which learners regulate progress toward their goals (Zimmerman and Moylan, 2009; Schunk and Zimmerman, 2011). The present findings extend this theoretical perspective to teachers’ AI-related professional learning. The distinction between the profiles was not attributable to a single dominant strategy; rather, stronger or more limited regulation appeared across multiple dimensions simultaneously. This pattern suggests that teachers’ capacity to regulate AI-related learning may be better understood as a configuration of mutually related regulatory processes than as a set of isolated competencies. This pattern also supports the value of a person-centered approach for revealing heterogeneity that may remain obscured when SRL dimensions are examined independently.

A second important finding concerns the association of AI experience and prior AI-related training with profile membership. Teachers with AI experience and those who had received AI-related training were more frequently represented among High Strategic Learners. Importantly, the small-to-moderate effect sizes observed for these associations suggest meaningful, but not deterministic, relationships. One possible explanation is that repeated engagement with AI provides teachers with opportunities to establish clearer learning goals, evaluate the usefulness of different tools, recognize gaps in their knowledge, and seek assistance when difficulties arise. Formal AI training may similarly provide structured opportunities to practice regulatory behaviors that can be more difficult to develop through unstructured exposure alone. Recent intervention research further supports the importance of structured professional learning opportunities. Tan et al. (2025b) found that a six-month professional development programme grounded in the Intelligent-TPACK framework significantly improved university teachers’ AI competency, suggesting that sustained and systematically designed learning opportunities can strengthen the knowledge and pedagogical competencies required for meaningful AI integration. This interpretation is broadly consistent with previous research emphasizing the relevance of AI familiarity, self-efficacy, perceived usefulness, and learning opportunities for teachers’ engagement with emerging technologies (Pokrivcakova, 2023; Chounta et al., 2022; Gökçe Tekin, 2024).

Nevertheless, these associations should not be interpreted as evidence that AI experience or training causes stronger self-regulation. Given the cross-sectional design of the study, the direction of the relationship cannot be established. An alternative explanation is that teachers who already possess stronger self-regulatory tendencies may be more inclined to explore AI technologies, participate in AI-related training, and actively seek opportunities to develop their competencies. It is also possible that the relationship is reciprocal, with greater self-regulation encouraging AI engagement and continued engagement providing additional opportunities to exercise self-regulatory strategies. Longitudinal and intervention-based research is therefore needed to clarify how AI experience, professional training, and self-regulated learning influence one another over time.

The findings concerning institutional support further suggest that AI-related self-regulation should not be conceptualized solely as an individual capability. Teachers regulate their professional learning within organizational environments that can facilitate or constrain opportunities for learning. Access to technical infrastructure, professional development opportunities, collegial assistance, and institutional guidance may provide conditions in which teachers can more effectively plan, monitor, and evaluate their AI-related learning. This interpretation is consistent with previous studies emphasizing institutional support as an important condition for successful technology integration (Lindner and Romeike, 2019; Cabero-Almenara et al., 2024; Moylan et al., 2025). Theoretically, this finding supports treating institutional support as an important contextual dimension of AI-related self-regulated professional learning rather than as an exclusively individual regulatory process.

From a professional learning perspective, teachers represented by the Limited Strategy Users profile may benefit from support that addresses both AI-related competencies and the regulatory processes involved in developing those competencies. This interpretation is consistent with previous research indicating that AI knowledge, perceived pedagogical value, and competence-related concerns may shape teachers’ engagement with AI technologies (Kalra, 2024; Nazaretsky et al., 2022; Hwang et al., 2020).

The absence of meaningful differentiation according to the demographic characteristics examined provides another important perspective on teacher learning in AI contexts. Age, professional experience, and school type did not significantly differentiate the identified profiles, while gender also showed little practical association with profile membership. Within the present sample, this pattern suggests that relatively stable demographic characteristics may be less informative for distinguishing AI-SRL profiles than teachers’ opportunities to engage with AI and participate in relevant professional learning. This interpretation is consistent with research suggesting that teachers’ digital competencies are associated with professional learning opportunities and technology-use experience rather than being reducible to age or professional seniority alone (Daher, 2025; Guan et al., 2025). These findings therefore caution against designing AI professional development on the assumption that particular demographic groups necessarily require more or less support.

Taken together, the findings extend current research on teachers’ engagement with AI in two important ways. First, whereas previous studies have largely emphasized attitudes, acceptance, literacy, or readiness (Kohnke et al., 2025; López-Costa et al., 2025), the present study shifts attention toward the processes through which teachers regulate their own AI-related professional learning. Second, the person-centered approach demonstrates that teachers with different combinations of regulatory strategies can be distinguished as meaningful learning profiles. The theoretical contribution therefore lies not simply in showing that SRL matters in AI contexts, but in demonstrating that multiple SRL dimensions can operate together as distinct configurations of professional learning. This perspective provides a bridge between self-regulated learning theory and the emerging literature on teachers’ AI-related professional development.

From a practical perspective, the profiles suggest the potential value of differentiated professional development. Limited Strategy Users may benefit from more structured support for goal setting, monitoring, reflection, and help seeking, whereas High Strategic Learners may benefit from greater autonomy, advanced AI-related learning opportunities, and collaborative experimentation. These profiles should not be viewed as fixed categories, but as empirically derived patterns that may help inform professional learning design.

## 6 Conclusions and implications

As artificial intelligence becomes increasingly integrated into education, understanding how teachers regulate their own professional learning is essential for supporting meaningful and sustainable AI adoption. Moving beyond approaches that treat teachers as a homogeneous group, this study demonstrates the value of examining AI-related professional learning through a person-centered self-regulated learning perspective.

The main finding of the study is the identification of two distinct AI-SRL profiles: High Strategic Learners and Limited Strategy Users. These profiles reflect broader configurations of task planning, self-monitoring, help seeking, institutional support, and self-evaluation rather than differences in a single learning strategy. AI experience and prior AI-related training were meaningfully associated with profile membership, whereas the demographic characteristics examined provided comparatively limited differentiation. Within the present sample, these findings suggest that AI-related learning experiences and opportunities may be more informative for distinguishing the identified profiles than the demographic characteristics examined.

Theoretically, the study extends self-regulated learning research into the context of teachers’ AI-related professional learning by demonstrating that multiple regulatory dimensions can operate together as distinct configurations within individuals. The person-centered approach complements traditional variable-centered research by revealing heterogeneity that may be obscured when individual SRL dimensions are examined separately. In addition, the role of institutional support highlights the importance of understanding AI-related self-regulation as a process shaped by both individual regulatory capacities and contextual learning opportunities.

From a practical perspective, the findings support a differentiated approach to teacher professional development. Limited Strategy Users may benefit from structured goal-setting, guided AI practice, explicit monitoring and reflection activities, accessible help-seeking opportunities, and stronger institutional scaffolding. High Strategic Learners may benefit from more autonomous and advanced AI learning opportunities, collaborative experimentation, and opportunities to share expertise with colleagues. Professional development initiatives should therefore consider not only what teachers need to learn about AI but also how they regulate the process of acquiring and updating AI-related competencies.

Future research should examine whether these profiles remain stable or change as teachers gain greater experience with AI. Longitudinal studies could clarify the direction of the relationships among AI experience, professional training, and self-regulated learning, while intervention studies could investigate whether targeted professional development facilitates movement toward more strategic learning configurations. Future studies should also replicate the identified profiles in larger and more diverse teacher populations and examine whether profile membership is associated with actual AI use, instructional practices, and student-related outcomes. Such research would provide a stronger basis for developing adaptive and evidence-informed professional learning systems for teachers in rapidly evolving AI-integrated educational environments.

## 7 Limitations

Despite its contributions, this study has several limitations that should be considered when interpreting the findings. First, the study was conducted with in-service teachers from different provinces of Türkiye. Although the sample included teachers with diverse demographic and professional characteristics, the cultural, institutional, and educational characteristics of the Turkish context may have influenced both teachers’ opportunities to engage with AI and the ways in which they regulate their professional learning. Consequently, the two AI-SRL profiles identified in this study should not be assumed to generalize directly to teachers in educational systems with different technological infrastructures, professional development structures, or institutional expectations. Future research should replicate the profile structure using larger and more diverse samples across countries and educational systems and examine whether similar AI-SRL configurations emerge across cultural and institutional contexts.

Second, the study relied on self-report measures of teachers’ AI-related self-regulated learning. Self-report instruments are useful for capturing teachers’ perceptions of their regulatory strategies, but they may be affected by social desirability, recall limitations, or discrepancies between perceived and actual behavior. As a result, the identified profiles represent teachers’ reported patterns of strategy use and may not fully correspond to how they regulate their learning during actual interactions with AI technologies. Future studies should combine self-report measures with behavioral and process-based indicators, such as learning analytics, AI-use logs, observational data, think-aloud protocols, or trace data, to examine whether reported AI-SRL profiles are reflected in teachers’ actual learning behaviors.

Third, the cross-sectional design limits conclusions regarding the temporal development and directionality of the observed relationships. Although AI experience and prior AI-related training were significantly associated with profile membership, the present data cannot establish whether experience and training contribute to stronger self-regulation, whether teachers with stronger self-regulatory tendencies are more likely to seek AI-related experiences and training, or whether these processes influence one another over time. Future longitudinal studies should examine transitions between AI-SRL profiles as teachers gain experience with AI, while intervention or experimental studies could investigate whether targeted professional development produces changes in specific self-regulatory strategies or profile membership.

Finally, the identification of teacher profiles was based primarily on Two-Step Cluster Analysis. Although formal resampling-based stability testing was not conducted, robustness was examined through comparison of alternative cluster solutions and a K-means sensitivity analysis. Nevertheless, cluster solutions remain data-dependent and may vary across samples or analytical approaches. Accordingly, the two profiles should be interpreted as empirically derived patterns within the present sample rather than as fixed or universal categories of teachers. Future research should replicate the analysis with independent samples and evaluate the reproducibility and stability of the identified profiles using formal resampling-based procedures and alternative person-centered methods, including Latent Profile Analysis.

## Data Availability

The raw data supporting the conclusions of this article will be made available by the authors, without undue reservation.

## References

[ref1] Al Darayseh A. (2023). Acceptance of artificial intelligence in teaching science: science teachers’ perspective. Comput. Educ.: Artif. Intell. 4:100132. doi: 10.1016/j.caeai.2023.100132, 42574925

[ref2] Alpsülün M. Vuran M. Balıkçı H. C. (2025) Öğretmenlerin yapay zekâ öğrenme süreçlerine yönelik öz-düzenlemeli öğrenme stratejileri ölçeğinin Türkçeye uyarlanması: Geçerlik ve güvenirlik çalışması Proceedings book of the 5th International Conference on Frontiers in Academic Research (ICFAR 2025) 340–348 Konya, Türkiye: All Sciences Academy.

[ref3] Balıkçı H. C. Alpsülün M. (2025). Turkish adaptation of artificial intelligence attitude Scale-4: investigating general AI attitudes through various factors. e-Kafkas J. Educ. Res. 12, 444–457. doi: 10.30900/kafkasegt.1509077

[ref4] Balıkçı H. C. Alpsülün M. Hayoğlu G. (2024). Determination of teachers’ perceptions of artificial intelligence concept: a metaphor analysis. Sakarya Univ. J. Educ 14, 179–193. doi: 10.19126/suje.1325153

[ref5] Bergman L. R. Magnusson D. (1997). A person-oriented approach in research on developmental psychopathology. Dev. Psychopathol. 9, 291–319. doi: 10.1017/S095457949700206X 9201446

[ref6] Cabero-Almenara J. Palacios-Rodríguez A. Loaiza-Aguirre M. I. Rivas-Manzano M. D. R. D. (2024). Acceptance of educational artificial intelligence by teachers and its relationship with some variables and pedagogical beliefs. Educ. Sci. 14:740. doi: 10.3390/educsci14070740

[ref7] Chounta I.-A. Bardone E. Raudsep A. Pedaste M. (2022). Exploring teachers’ perceptions of artificial intelligence as a tool to support their practice in Estonian K–12 education. Int. J. Artif. Intell. Educ. 32, 725–755. doi: 10.1007/s40593-021-00243-5

[ref8] Cohen J. (1988). Statistical power Analysis for the Behavioral Sciences. 2nd Edn. Hillsdale, NJ: Lawrence Erlbaum Associates.

[ref9] Creswell J. W. Creswell J. D. (2017). Research design: Qualitative, Quantitative, and Mixed Methods Approaches. Thousand Oaks, CA: SAGE Publications.

[ref10] Daher R. (2025). Integrating AI literacy into teacher education: a critical perspective paper. Discov. Artif. Intell. 5:217. doi: 10.1007/s44163-025-00475-7

[ref11] Delcker J. Heil J. Ifenthaler D. (2025). Evidence-based development of an instrument for the assessment of teachers’ self-perceptions of their artificial intelligence competence. Educ. Technol. Res. Dev. 73, 115–133. doi: 10.1007/s11423-024-10418-1

[ref13] Gökçe Tekin Ö. (2024). Factors affecting teachers’ acceptance of artificial intelligence technologies: analyzing teacher perspectives with structural equation modeling. Instructional Technology and Lifelong Learning 5, 399–420. doi: 10.52911/itall.1532218

[ref14] Greene J. A. Bernacki M. L. Hadwin A. F. (2023). “Self-regulation,” in Handbook of Educational Psychology, eds. Schutz P. A. Muis K. R. (New York, NY: Routledge), 314–334.

[ref15] Guan L. Zhang Y. Gu M. M. (2025). Pre-service teachers preparedness for AI-integrated education: an investigation from perceptions, capabilities, and teachers’ identity changes. Comput. Educ.: Artif. Intell 8:100341. doi: 10.1016/j.caeai.2024.100341

[ref16] Hair J. F. Black W. C. Babin B. J. Anderson R. E. (2019). Multivariate data analysis. 8th Edn. Andover, Hampshire: Cengage.

[ref17] Howard M. C. Hoffman M. E. (2018). Variable-centered, person-centered, and person-specific approaches: where theory meets the method. Organ. Res. Methods 21, 846–876. doi: 10.1177/1094428117744021

[ref18] Hwang G.-J. Xie H. Wah B. W. Gašević D. (2020). Vision, challenges, roles and research issues of artificial intelligence in education. Comput. Educ.: Artif. Intell 1:100001. doi: 10.1016/j.caeai.2020.100001, 42574925

[ref19] Kalra R. (2024). Exploring teachers’ perceptions toward the integration of AI tools in the language classroom. NIDA J Lang Commun 29, 21–36. Available at: https://so04.tci-thaijo.org/index.php/NJLC/article/view/280606

[ref20] Kauffman D. F. (2004). Self-regulated learning in web-based environments: instructional tools designed to facilitate cognitive strategy use, metacognitive processing, and motivational beliefs. J. Educ. Comput. Res. 30, 139–161. doi: 10.2190/ax2d-y9vm-v7px-0tad

[ref21] Kaufman L. Rousseeuw P. J. (1990). Finding Groups in data: An Introduction to Cluster Analysis. New York: John Wiley & Sons.

[ref22] Kaya I. Meylani R. (2026). AI literacy for self-regulated learning among pre-service teachers: development and validation of a scale of ethical use, monitoring, motivation, regulation, and evaluation. Front. Psychol. 17:1948694. doi: 10.3389/fpsyg.2026.1948694, 42724067 PMC13558209

[ref23] Kohnke L. Zou D. Ou A. W. Gu M. M. (2025). Preparing future educators for AI-enhanced classrooms: insights into AI literacy and integration. Comput. Educ.: Artif. Intell. 8:100398. doi: 10.1016/j.caeai.2025.100398, 42574925

[ref24] Lindner A. Romeike R. (2019). Teachers’ perspectives on artificial intelligence. In Jasutė E. Pozdniakov S. (Eds.), ISSEP 2019: 12th International Conference on Informatics in Schools: Situation, Evolution and Perspectives, local Proceedings (pp. 22–29).

[ref25] López-Costa M. Donate-Beby B. Cabrera-Lanzo N. Maina M. F. (2025). Understanding AI adoption among secondary education teachers: a PLS-SEM approach. Comput. Educ. Artif. Intell. 8:100416. doi: 10.1016/j.caeai.2025.100416

[ref26] Ma S. Lei L. (2024). The factors influencing teacher education students’ willingness to adopt artificial intelligence technology for information-based teaching. Asia Pac. J. Educ. 44, 94–111. doi: 10.1080/02188791.2024.2305155

[ref27] Moylan R. Code J. O’Brien H. (2025). Teaching and AI in the postdigital age: learning from teachers’ perspectives. Teach. Teach. Educ. 153:104851. doi: 10.1016/j.tate.2024.104851

[ref28] Nazaretsky T. Ariely M. Cukurova M. Alexandron G. (2022). Teachers’ trust in AI-powered educational technology and a professional development program to improve it. Br. J. Educ. Technol. 53, 914–931. doi: 10.1111/bjet.13232

[ref29] Norušis M. J. (2011). IBM SPSS Statistics 19 Guide to Data Analysis. Upper Saddle River, NJ: Pearson Prentice Hall.

[ref30] Nunnally J. C. Bernstein I. H. (1994). Psychometric Theory. 3rd Edn. New York, NY: McGraw-Hill.

[ref31] OECD (2023). OECD digital Education Outlook 2023: Towards an Effective Education Ecosystem. Paris: OECD Publishing. doi: 10.1787/c74f03de-en

[ref32] Pokrivcakova S. (2023). Pre-service teachers’ attitudes towards artificial intelligence and its integration into EFL teaching and learning. J. Lang. Cult. Educ. 11, 100–114. doi: 10.2478/jolace-2023-0031

[ref33] Randi J. (2004). Teachers as self-regulated learners. Teach. Coll. Rec. 106, 1825–1853. doi: 10.1177/016146810410600908

[ref34] Rousseeuw P. J. (1987). Silhouettes: a graphical aid to the interpretation and validation of cluster analysis. J. Comput. Appl. Math. 20, 53–65. doi: 10.1016/0377-0427(87)90125-7

[ref35] Schunk D. H. Zimmerman B. J. (2011). Handbook of Self-Regulation of Learning and Performance. New York: Taylor & Francis.

[ref36] Sumandal A. H. (2023). Teachers’ self-efficacy with artificial intelligence (AI) based educational tools. Ignatian International Journal for Multidisciplinary Research 1, 1–10. doi: 10.5281/zenodo.10002206

[ref37] Tan X. Cheng G. Ling M. H. (2025a). Artificial intelligence in teaching and teacher professional development: a systematic review. Comput. Educ. Artif. Intell. 8:100355. doi: 10.1016/j.caeai.2024.100355, 42574925

[ref38] Tan X. Cheng G. Ling M. H. (2025b). Enhancing teachers’ AI competency: a professional development intervention study based on intelligent-TPACK framework. Comput. Educ. Artif. Intell. 9:100521. doi: 10.1016/j.caeai.2025.100521, 42574925

[ref39] Temur S. (2025). Yapay zekâ ve eğitim: Güncel uygulamalar ve gelecek perspektifleri. Trakya Üniversitesi Sosyal Bilimler Dergisi 27, 162–178. doi: 10.26468/trakyasobed.1539210

[ref40] UNESCO (2023). Guidance for Generative AI in education and research. Available online at: https://unesdoc.unesco.org/ark:/48223/pf0000386693 (Accessed April 28, 2026).

[ref41] Zelazo P. D. (2014). “Self-reflection and the development of consciously controlled processing,” in Children’s Reasoning and the mind, eds. Peter M. Riggs K. J. (Hove, UK: Psychology Press), 169–189.

[ref42] Zhang W. Ning Y. Yu N. Yao D. Wu H. Wu J. . (2025). Development and validation of the artificial intelligence self-regulated learning scale (AI-SRLS) for teachers. Educ. Inf. Technol. 30, 22989–23013. doi: 10.1007/s10639-025-13670-x

[ref43] Zimmerman B. J. Moylan A. R. (2009). “Self-regulation: where metacognition and motivation intersect,” in Handbook of Metacognition in Education, eds. Hacker D. J. Dunlosky J. Graesser A. C. (New York, NY: Routledge), 299–315.

